# Intussusception in the Setting of an Ulcerative Colitis Flare

**DOI:** 10.1155/2022/3559464

**Published:** 2022-05-30

**Authors:** Varag Abed, Alexis Faber, Cristina Jageka, Ryan Goleniak, Raef Fadel

**Affiliations:** Henry Ford Health System, 2799 W Grand Blvd, Detroit, MI 48202, USA

## Abstract

Intussusception is an extraordinary cause of acute abdomen in adults and has been defined as the telescoping of a bowel segment into the lumen of an adjacent segment. A 43-year-old female presented to our hospital's emergency department (ED) with 10+ episodes of bloody diarrhea per day, left-sided abdominal pain, and the inability to tolerate oral intake for one month. She was initially diagnosed with ulcerative colitis (UC) ten years ago and is currently on mesalamine oral and enema therapy. She presented to our gastroenterology clinic two weeks after the beginning of her flare and was started on prednisone 40 mg daily. This did not improve her symptoms, and she presented to the ED two weeks later. She underwent a computed tomography (CT) abdomen/pelvis which revealed intussusception in the left hemiabdomen with no definite lead point measuring 5.6 cm in the craniocaudal dimension with pneumatosis and no evidence of bowel obstruction. There were no other significant laboratory abnormalities. Acute care surgery was consulted and suggested obtaining a CT enterography for further evaluation which showed spontaneous resolution of intussusception with no evidence of pneumatosis, portal venous gas, or intraperitoneal free air. She reports that following oral contrast intake, she “felt movement and relaxation” in her abdomen with substantial pain relief. Infectious workup was negative, and therapy was initiated with intravenous steroids. In conclusion, intussusception has been very rarely reported in patients with UC with the most common treatment being surgical resection. However, conservative management in the absence of bowel obstruction can be attempted.

## 1. Introduction

Intussusception is a very rare cause of acute abdomen in adults and has been defined as the telescoping of a bowel segment into the lumen of an adjacent segment [1, 2]. In patients with ulcerative colitis (UC), only seven cases of concurrent intussusception have been reported in the literature [2–8]. Abdominal pain is usually the presenting sign with most patients requiring surgical resection [9]. In this case report, we describe a patient with an active UC flare who developed symptomatic abdominal pain and intussusception that resolved following oral contrast administration at a university teaching hospital.

## 2. Case Report

**Figure 1:**
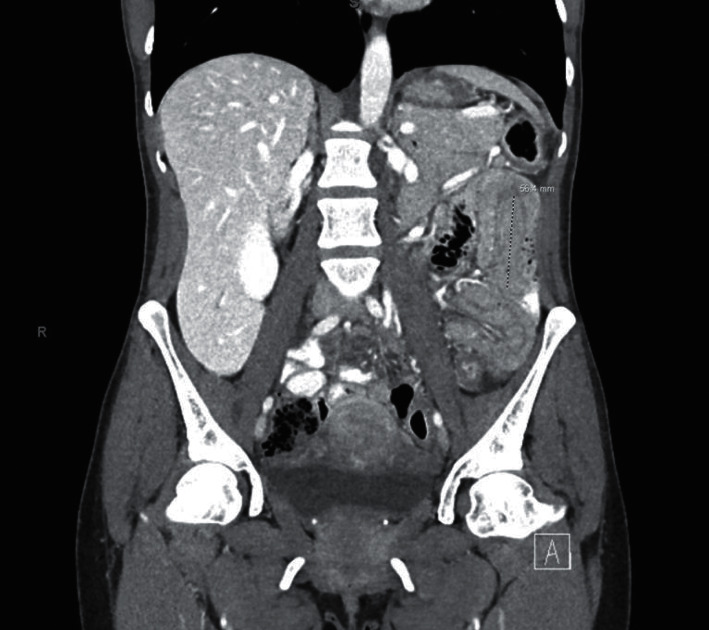
CT scan showing intussusception in the left hemiabdomen.

**Figure 2:**
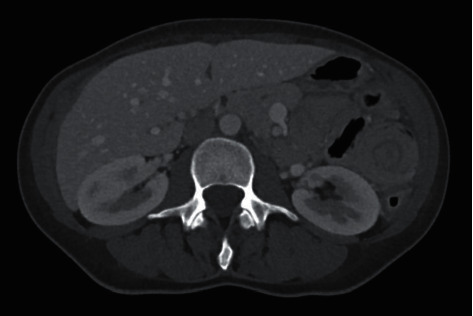
CT scan showing the “Target Sign.”

A 43-year-old female presented to our hospital's emergency department (ED) with 10+ episodes of bloody diarrhea per day, left-sided abdominal pain, and the inability to tolerate oral intake for one month. She was initially diagnosed with ulcerative colitis ten years ago and is currently on mesalamine oral and enema therapy. Most recent colonoscopy was done in 2020 and revealed extensive colitis of the transverse, descending, and sigmoid colon with active proctitis. She usually has two to three flares a year where she manages them by herself through diet modification and lifestyle changes (liquid diet and small meals with lean proteins), but this time her symptoms continued worsening causing her to come to the hospital. She presented to our gastroenterology (GI) clinic two weeks after the beginning of this current flare and was started on prednisone 40 mg daily. They recommended discontinuing the mesalamine enema due to excess diarrhea, which she did, but she inadvertently also stopped taking her oral mesalamine. The prednisone did not improve her symptoms, as she was still having 10+ bloody bowel movements per day and severe left-sided abdominal pain described as a constant throbbing sensation with swelling and warmth overlying the skin. She presented to our ED two weeks later and was found to have an elevated c-reactive protein of 4.4 mg/dL, erythrocyte sedimentation rate of 51 mm/Hr, blood pressure of 164/101 mmHg, pulse of 107 with a soft, nondistended, tender abdomen, and dry mucous membranes. A Truelove and Witts severity index scoring was utilized, resulting in a severe classification. Also, the Montreal classification for inflammatory bowel disease scoring was rated as E3 S3. She underwent a computed tomography (CT) abdomen/pelvis which revealed intussusception in the left hemiabdomen with no definite lead point measuring 5.6 cm in the craniocaudal dimension with pneumatosis and no evidence of bowel obstruction. Diffuse colorectal wall thickening, mucosal hyperenhancement, and fat stranding were also seen suggesting proctocolitis (Figures 1 and 2). There were no other significant laboratory abnormalities. Acute care surgery was consulted and suggested obtaining a CT enterography for further evaluation, which showed spontaneous resolution of intussusception with no evidence of pneumatosis, portal venous gas, or intraperitoneal free air. She reports that following oral contrast (Breeza) intake, she “felt movement and relaxation” in her abdomen with substantial pain relief. She was subsequently transferred to the medicine floor with a GI consultation for management of her UC flare. Infectious workup was negative, and therapy was initiated with intravenous steroids. Her hospital stay was uneventful as she was discharged five days later on an oral prednisone taper with GI follow-up.

## 3. Discussion

To the best of our knowledge, there have only been seven cases of patients with UC complicated with intussusception in the literature (Table 1). In the adult population, neoplastic lead points are the most common cause of intussusception [10], but surprisingly, none of the previous reported cases have been found to have an underlying neoplasm. Tanabe et al. [2] hypothesized that this could be due to the frequent colonoscopies patients with UC receive. The most common cause has been inflammatory polyps with four of the previous seven case reports citing them as the underlying etiology, and the most common treatment was surgical resection in five of the seven previous cases (Table 1).

CT scan has been regarded as the modality of choice for diagnosing intussusception [11]. Due to the clinical progression of our patient, we believe intussusception was the likely cause of her UC flare which was found on initial CT. Most studies of intussusception have been performed on children, as they are much more likely to receive the diagnosis than adults. Nonsurgical conservative management for intussusception consists of barium, water-soluble contrast media, water, electrolyte solutions, or air [12]. Our patient had resolution of her intussusception with the oral contrast used for CT enterography, which to our knowledge has not been reported in the literature. However, we cannot definitively determine this to be the reason as the intussusception could have been transient which resolved spontaneously [13]. Notwithstanding, resolution of intussusception has been seen previously after oral gastrografin ingestion [14].

In conclusion, intussusception has been previously reported with patients with UC with the most common treatment being surgical resection. However, based on our findings, conservative management with oral contrast could be attempted before surgical resection in adults in the absence of bowel obstruction.

## Figures and Tables

**Table 1:** Reported cases of concurrent intussusception and ulcerative colitis.

Author	Year	Age	Gender	Location	Etiology	Symptoms	Treatment
Tanabe et al. [2]	2020	18	Male	Transverse colon	Inflammatory polyps	None	Surgical resection
Burchard and Thomay [3]	2018	39	Female	Appendix	Appendicitis	Abdominal pain	Surgical resection
Coghlan et al. [4]	2010	35	Female	Transverse colon	CMV infection	Abdominal pain	Conservative medical approach
Davey et al. [5]	2020	42	Male	Hepatic flexure	Appendicitis	Abdominal pain	Surgical resection
Esaki et al. [6]	2009	27	Male	Hepatic flexure	Inflammatory polyps	Abdominal pain	Enema reduction
Forde et al. [7]	1978	22	Male	Transverse colon	Inflammatory polyps	Abdominal pain	Surgical resection
Maldonado et al. [8]	2004	27	Male	Splenic flexure	Inflammatory polyps	Abdominal pain	Surgical resection
Current case	2022	43	Female	Left hemiabdomen	Unknown	Abdominal pain	Oral contrast reduction

Table adapted from Tanabe et al. [2].

## Data Availability

No data were used to support this study.
